# Artificial intelligence application in counselling practices. A multigroup analysis of acceptance and awareness using gender and professional rank

**DOI:** 10.3389/fdgth.2024.1414178

**Published:** 2025-03-19

**Authors:** Usani Joseph Ofem, Pauline Mbua Anake, Cyril Bisong Abuo, James Omaji Ukatu, Eugene Onor Etta

**Affiliations:** ^1^ Department of Educational Foundations, Alex Ekwueme Federal University Ndufu-Alike, Abakaliki, Ebonyi, Nigeria; ^2^ Department of Guidance and Counselling, University of Calabar, Calabar, Nigeria; ^3^ Department of Criminology, Alex Ekwueme Federal University Ndufu-Alike, Abakaliki, Ebonyi, Nigeria; ^4^ Department of Public Administration, Federal Polytechnic Ugep, Ugep, Cross River, Nigeria

**Keywords:** awareness, acceptance, artificial intelligence, multi-group analysis, gender, professional rank

## Abstract

**Introduction:**

Artificial intelligence (AI) has emerged as a transformative tool in various professional domains, including counselling, where it offers innovative ways to enhance service delivery and client outcomes. Despite its potential, research on AI in counselling practices often focuses on its technical applications, with limited attention to the interplay between awareness, acceptance, and application. This study analyses how professional counsellors apply artificial intelligence in counselling practices using the nexus between awareness and application through acceptance of AI with gender and professional rank as group.

**Method:**

A total of 5,432 professional counsellors were selected for the study. Data collection was conducted online to ensure a wide reach. The research instruments underwent validity checks, demonstrating high content and factorial validity. Convergent and discriminant validity were confirmed using the Average Variance Extracted (AVE) and Fornel-Larcker criterion.

**Results:**

The findings revealed that professional counsellors exhibited high levels of awareness, acceptability, and application of AI in their counselling practices. Acceptance played a positive mediating role in the relationship between awareness and application. However, male practitioners and professors displayed stronger awareness, acceptance, and application of AI tools compared to their counterparts.

**Conclusion:**

The study highlights the significant role of acceptance in bridging awareness and application of AI in counselling practices. It underscores the importance of addressing gender and professional rank disparities to ensure equitable adoption and utilization of AI tools. The findings offer valuable insights for policymakers in promoting the integration of AI in counselling to enhance professional practices.

## Introduction

Counseling services have been integral in addressing mental health issues, offering support, and facilitating personal development for individuals worldwide. However, the increasing demand for mental health services often surpasses the available resources, leading to significant gaps in accessibility and affordability. In response to these challenges, researchers and practitioners have turned to technology, particularly Artificial Intelligence (AI), to augment traditional counseling approaches and enhance service delivery. Artificial Intelligence, defined as the simulation of human intelligence processes by machines, has seen rapid advancements in recent years, offering new possibilities in various fields, including healthcare and mental health services (1). In the realm of counseling, AI technologies present innovative solutions to complement traditional therapeutic practices, expand outreach, and improve the overall quality of care (2).

Currently, the integration of artificial intelligence (AI) in counseling activities represents a paradigm shift in mental health care delivery, offering innovative solutions to address various challenges faced by traditional counseling approaches. AI technologies, including machine learning algorithms, natural language processing (NLP), and chatbots, have been increasingly utilized in counseling settings to enhance accessibility, efficiency, and effectiveness of mental health services (3). The primary benefit been that it improves accessibility, to mental support programmes in remote areas, thereby overcoming geographical issues and limitations that have been placed due to transportation cost and social stigmatization (4). Virtual counseling platforms equipped with AI-powered chatbots, or virtual agents offer round-the-clock support, allowing individuals to receive immediate assistance and guidance whenever needed (5, 6). Similarly, AI in counselling facilitates the delivery of personalized and tailored intervention based on the individuals' unique needs, preferences, and characteristics. Machine learning algorithms analyze vast amounts of data, including user interactions, behavioral patterns, and self-reported symptoms, to generate personalized recommendations and interventions (7). By adapting interventions to individual needs, AI-driven counseling platforms can enhance engagement, motivation, and outcomes of therapeutic interventions (8). Vaidyam et al. (9) reported that ChatGPT an AI tool can handle multiple interactions simultaneously, providing immediate responses and support to many users concurrently without being constrained by limited human resources or scheduling constraints (10–17).

In Nigeria, despite the growing interest in AI-driven counseling interventions, there is limited research exploring the intersection of gender and professional rank in shaping the utilization and perceptions of these technologies within the counseling context. Additionally, the COVID-19 pandemic has exacerbated these challenges, highlighting the urgent need for innovative and scalable solutions to address mental health needs remotely. Within this context, there is a notable gap in the utilization of Artificial Intelligence (AI) in counseling activities in Nigeria. While AI technologies hold promise for expanding access to mental health support and enhancing service delivery globally, their application and effectiveness within the Nigerian context remain largely unexplored. Limited research exists on the awareness, acceptability, application of AI-driven counseling interventions tailored to the socio-cultural and infrastructural realities of Nigeria.

Research suggests that gender may impact individuals' attitudes and preferences towards technology-mediated interventions (18). Women, for instance, may demonstrate greater openness to seeking support from AI-powered platforms, perceiving them as non-judgmental and accessible (19). Conversely, men may exhibit more skepticism or resistance towards AI counseling, preferring traditional face-to-face interactions (19). Understanding these gender differences is essential for tailoring AI interventions to effectively engage and meet the needs of diverse user populations. Professional rank within the counseling field, such as clinicians, counsellors in training, and support staff, may also influence the adoption and utilization of AI technologies. Experienced clinicians may view AI as a supplement to their existing skills and expertise, leveraging it to enhance the quality and efficiency of their services (20). In contrast, novice counselors or support staff may perceive AI as a substitute for human interaction, raising concerns about job displacement or devaluation of counseling skills (20). Examining the attitudes and experiences of different professional ranks towards AI in counseling can inform training and implementation strategies to maximize its integration and impact within clinical settings.

Previous studies have explored the role of AI in education, focusing mainly on instruction, assessment, and administration, among others. The Chubb et al. (21) study was focused on AI research, which relied on thematic areas to identify the factors that affect the utilisation of AI tools by university staff. Even though this study does not focus on counselling, it provided useful insight, like ethical considerations in the limitations faced by most professionals. Other studies found that even though most academic staff are aware of AI tools in education, studies have not focused on how they are applied in counselling practices (22). In another instance, researchers have stated that most people have a very patchy understanding of how these tools are applied across populations, and this is often influenced by several factors, such as the media (23–25). More so, Bingimlas (26) averred that the application of AI can be influenced by awareness and acceptance of technology by the staff when it is perceived as useful and easy to use. Hiltye et al. (27) further noted that, even though AI is new to most people *a priori*, “AI brings opportunities to involve rural areas that have insufficient medical resources, better patient response, and save time for clinicians in the U.S.”.

However, it is unclear whether AI, with its diverse tools, has been applied in Nigerian professional counselling sessions. To the best of our knowledge, studies of AI application in professional counselling in Nigeria have not been adequately examined. This may not be unconnected to the fact that Nigeria is still grappling with the problems of infrastructural decay, lack of access to ICT materials, ethical issues, and a lack of skills to handle AI tools in counselling sessions, among others (28). One study that has attempted to examine the application of AI in mental health includes Zhou et al. (29). This study appears to be the only one like what AI tools can be used for in mental health treatments. Yet, this study was not conducted in Nigeria. This study seeks to address this gap by examining the awareness through the acceptability of AI in the utilization of AI-powered counseling interventions tools in Nigeria. By leveraging AI technologies, such as chatbots and virtual agents, this research aims to overcome understand this nexus to provide scalable mental health support to underserved populations. Furthermore, by investigating the awareness through acceptability of AI-driven counseling among Nigerians and exploring potential utilization, this study aims to ensure the awareness and applicability of these interventions within the local context. Similarly, there is a need for decision-making that is based on empirical evidence to develop policies that are timely and promote the professional development of counsellors in line with new global practices. This study, therefore, looks at the interlink between awareness and acceptance of AI tools in counselling practices through acceptability, using gender and professional rank differences.

## Literature review

### Studies on awareness of AI

The emergence of AI in counselling has transformed professional practice in that it provides better support to clients using various techniques (11, 12). Most counsellors are increasingly recognising the effect of AI tools on the discharge of their professional responsibilities. It has been documented that AI tools in counselling can assist in providing basic counselling support as well as carrying out initial assessments through virtual therapy assistants (VTA) (30), understanding clients problems and tailoring their interventions using sentimental analysis that is possible through AI algorithms (28), developing professional treatment plans that can both recognise the needs and rights of the clients using past records and experiences, and monitoring the progress of the clients (31).

Research on professional counsellors' awareness of AI tools is inadequate. Most of the studies on awareness of AI tools in higher institutions have been mostly on staff and not on counsellors. However, staff who are in higher education may be aware of the availability of these tools for enhancing various academic tasks such as academic research writing (11, 12), assessment (32), and administration (33), among others. The integration of AI tools in counselling offers the profession an efficient means with which traditional practices that are adjudged to be cumbersome, and complex can be made easy (34). Most researchers have acknowledged the fact that although most counsellors may be aware of the potentials of AI in counselling, their non-application of these tools may be due to ethical concerns, privacy issues, and potential bias in AI algorithms, which of course may require that the counsellor possess strong digital literacy skills (35).

A recent study by Stina (36) revealed that AI is impactful in career guidance and holds strong prospects for the discharge of professional responsibilities. In another study, Gado et al. (37) noted that knowledge of AI, attitude towards AI, and perceived usefulness were the reasons why psychology students accepted using AI. Information on awareness is limited, and this is not good for policymaking. To the best of the researcher's knowledge, research on this topic is limited, and these variables as conceptualised here may not have been examined in guidance literature. Given the relevance of digitalization in counselling practices, it is imperative that professional counsellors are aware of the potential benefits and risks that are attached to the engagement of any of the tools that are identified as useful in counselling activities. Thus, the following hypothesis were made.

H1a Extent of awareness of AI among professional counsellors is not high.
H1b Awareness does not significantly application of AI in counselling practices.
H1c Awareness does not significantly predict acceptability of AI among counsellors.
H1d Acceptability of AI does not mediate the relationship between awareness and application of AI in counselling practices.

### Studies on acceptability of AI tools

The acceptance of AI is necessary in counselling because of the ethical issues involved that hinder most people from attempting to work in that direction. The acceptability of AI is based on different contexts. The relevance of AI makes many users benefit from its usage and, thus, accept it for different purposes. However, there are cases of low acceptance, and this may likely decrease the number of tools that are engaged in their practices to the detriment of the client (38, 39). Thus, technological acceptance is a choice, and it is based on what the individual presumes can be done with such devices, the accuracy with which the device can be used to make firm decisions, and comfortability in developing plans for the clients (40). Where the individual perceives that such a facility can be useful or manipulated easily, the tendency for such acceptance can be very high (41). It is imperative to examine counsellors' acceptance since their work requires more humans that must be handled with care. Most of the counsellors are aware that confidentiality principles, informed consent, and beneficence are paramount in their work. Thus, whatever technology is to be employed must align with global best practices. The theory of technological acceptance model by Davis (42, 43), which measures the propensity for a tool to be accepted or rejected, is mostly used in studies that involve the acceptability of technology.

Some previous studies have attempted to examine the factors that influence acceptance of AI (44). Most of the studies that were conducted on technological acceptance were not in relation to counselling practices. For instance, Gado et al. (37) found that AI acceptance by psychology students is based on their perceived usefulness, attitude towards technology, and ease of se. This study, though it provided insight on factors that may influence acceptance, does not provide a link between acceptance and application by our population of interest.

H2a Extent of acceptability of AI among professional counsellors is not high.
H2b Acceptability does not significantly predict application of AI in counselling practices.

### Studies on application of AI in counselling practices

The varieties of problems that clients in school face are diverse, and AI offers the opportunity to address these issues (personal-social, educational, and vocational) with its tools (45–47). This is because AI has the capacity to function in a human-like manner (48). One of the recent studies by Doraiswamy et al. (49) found that mental health professionals believe in the application of AI, but such tools cannot replace the human component in the services they render to clients. Bickman (45) reported that most of the AI tools, like the Chatbot, offer various opportunities for interacting with clients and obtaining information that can be used by the counsellor to identify areas of need and develop intervention programmes that can aid in suggesting solutions and treatment packages that can benefit the clients.

The application of AI in counselling and mental health services is done with facilities that can interact, programme, and respond to queries and questions. It also consists of tools that can detect and predict various conditions through screening and, thus, make clinical decisions that human beings ordinarily cannot make. This is not to say that the application of AI in counselling practices will take up the functions of human counsellors (50). Similarly, Ellie digital avatar as an AI application tool is known for self-assessment and those struggling with depression (51). Others are the BioBase app, Woebot, and Elomia app, which are useful in handling cases of anxiety when it is still under control (52, 53).

Reports have shown that the use of these AI apps has demonstrated excellent results in mental health practices (54, 55). However, while AI has been used increasingly in other areas in the educational sector, its applicability in counselling practices is limited, and studies (56) that empirically provide this result are few or not available to the best of the researchers knowledge in Nigeria. In fact, it has also been argued that it is not the availability of the app that matters, but the application of these apps on various platforms for identification and treatment of psychological and social issues that the counselling profession is aimed t. Similarly, Seneviratne et al. (57) have noted that the application of AI in counselling practices can be likened to the “elephant in the room”. It is therefore expedient that the application of these tools by professional counsellors be examined to facilitate policymaking as well as help counsellors increase the efficiency and effectiveness of their services through AI applications; thus, it was hypothesised that:

H3a Professionals' extent of application of AI in counselling practices is not significantly high.

### Studies on demographic attributes

Demographic attributes in the context of this study are attributes that define the nature of the respondents in terms of gender, age, professional experience, professional ranks, and marital status, among others. Researchers focusing on these attributes have been a thing of concern because it is always difficult to decipher how a particular group of respondents are responding to a phenomenon. In the context of AI, which is a technological innovation, various studies have hitherto been carried out using groups like the age and gender of respondents (58–60). The issue of gender and technology has not been concluded in the literature, and there are disparities of findings even to this day (11, 12, 58, 61) However, other attributes, especially as it concerns the professional counsellors rank behaviour towards AI, have been underexplored in the literature. Professional rank describes the occupational status or level one has attained in the counselling profession, probably due to contributions, promotion, and years of ervice. Virtually all individuals differ in skills, ICT competence, and knowledge due to their height in the profession, and one expects that they might have been exposed to training and programmes related to AI through conferences and workshops, which provide them with the opportunity to be more aware of, accept, and apply these tools in the profession. For example, research has shown that higher education students who are doctorate and graduate students are more aware and have a better utilisation of AI in academic research (11, 12). This situation may be applicable to professionals with higher ranks in counselling practice. For example, Kleiman et al. (62) noted that given that senior professionals may have attended programmes related to ICT and handled diverse cases, which are more stressful, their awareness of these tools may have facilitated their acceptability and further application in their practices.

Nigeria, like other African countries, is still evolving with AI studies, and this contributes to the paucity of materials that have addressed studies of this magnitude. Limited infrastructure, a lack of expertise, and limited facilities may have hindered studies of this nature from being executed. The recent study by Syed and Al-Rawi (63) on perception, awareness, and opinion towards AI was focused more on descriptive and provided information on how AI was perceived by respondents who were not even professional counsellors. It is true that professionals still utilise traditional methods such as face-to-face interaction and the use of pen and paper in counselling, among others. The rationale is that most universities in Africa still use traditional methods in their counselling practices. “The level of digital materials that are necessary and required for full application of AI is not yet available, and teachers too may not be aware of the diverse AI tools that can facilitate quality and efficient outcomes” (11, 12). It is imperative that studies of this nature be carried out to provide a basis for policy development. It was hypothesis that.

H4a The direct effect of awareness on acceptability of AI tools in counselling practices is not significantly different based on gender.
H4b The direct effect of awareness on application of AI tools in counselling practices is significantly not different based on gender.
H4c The mediating effect of acceptability on application of AI tools in counselling practices is significantly not different based on gender.
H4d The direct effect of acceptability on application of AI tools in counseling practices is significantly not different based on gender.
H4e The direct effect of awareness on acceptability of AI tools in counselling practices is significantly not different between counsellors who are between within professional rank.
H4f The direct effect of awareness on application of AI tools in counselling practices is significantly not different based on professional ranks.
H4g. The direct effect of acceptability on application of AI tools in counseling practices is significantly not different based on professional rank.
H4h. The mediating effect of acceptability on application of AI tools in counselling practices is significantly not different based on professional rank.

**Figure 1:**
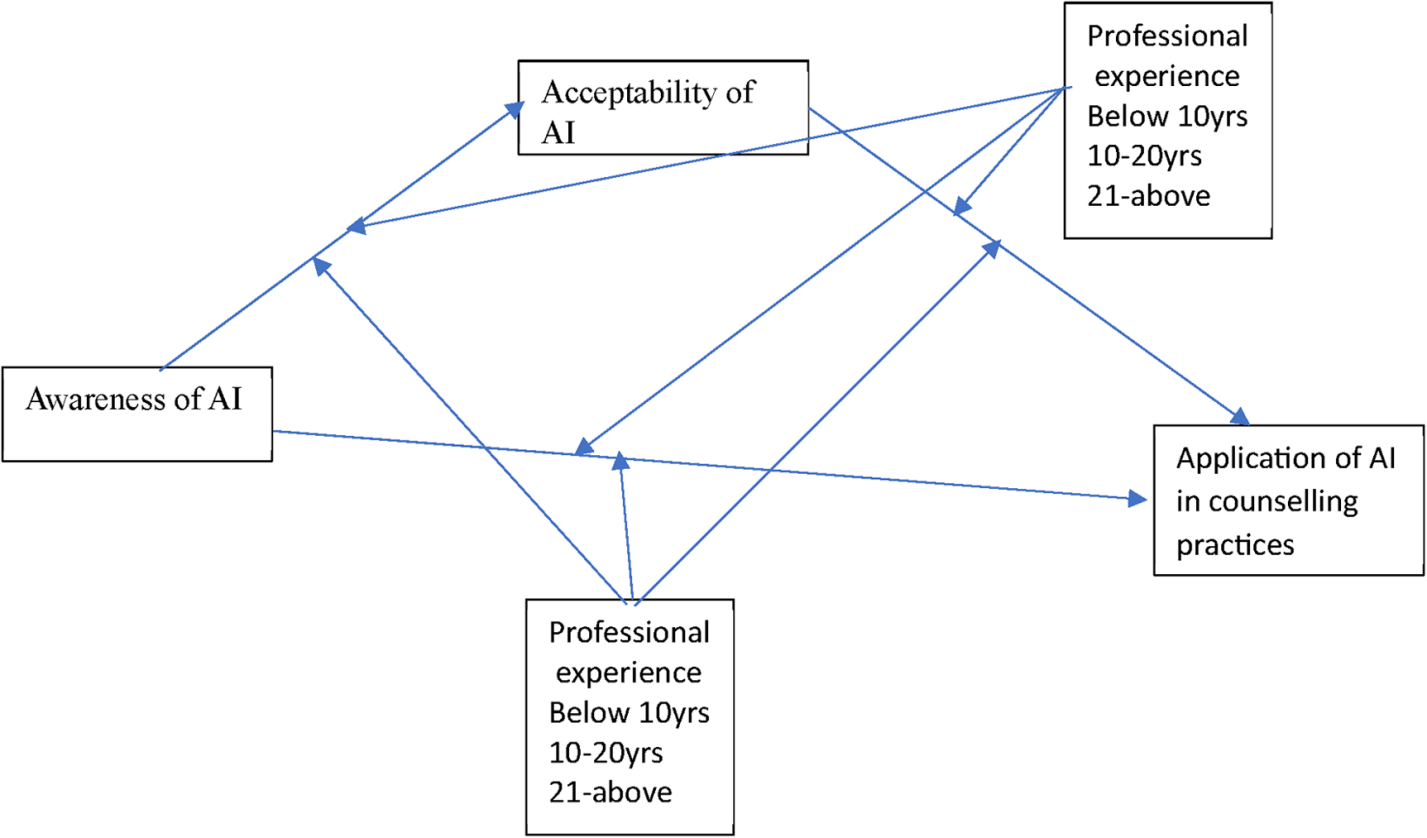
Conceptual framework on the linkages between awareness, acceptability, and applicability of AI on counselling practices with groups variations.

Conceptually, the linkages are represented in Figure 1 that shows how awareness is presumed to relate with acceptability of AI which in turn influences the applicability of AI in counsellor taking into cognizance the differential effect of professional experience and ranks of educational counsellors.

## Methodology

The study was a quantitative cross-sectional survey following the positivists research paradigm that utilizes various instruments in data collection at a particular point in order to have a nuanced understanding of the interlinkages of the variables of the study population was made up of 6,721 registered professional counsellors with the Counselling Association of Nigeria (CAN) from 76 public universities in Nigeria who have laptops, android, or iPhone. A total of 5,432 professionals were selected for the study through purposive sampling techniques. Purposive sampling technique was applied because of the difficulties associated with random selection and the researchers have knowledge of the characteristics of the respondents of the study. The demographic attributes of the population given as: 2,182 (40.17%) are males while 3,250 (59.83%) are females. 972 (17.89%) are single, 3,889 (71.59%) are married while 972 (17.89%) are divorced or separated. Similarly, 1,410 (25.96%) are within the rank of Asst Lect-Lect II, 2,872 (52.87%) are within the ranks of Lect I -Snr lecturer while 1,150 (21.17)are professors.

### Measures and instrument

There are five measures that are used in this study: awareness, acceptability, application of AI in counselling practices, professional experience, and ranks. Operationally, professional experience is measured using three categories based on the number of years that one has put into the profession, such as those below 10 years, 10–20 years, and 21 years and older. Professional rank is conceptualised as the status a counsellor has attained by virtue of inputs, years, and contributions to the field. Thus, respondents indicated their status by ticking any of the three categories provided as follows: Asst. Lect—Lect II, Lect I—Snr. Lect, and Professor. Awareness of AI refers to the knowledge and understanding that a counsellor has about the existence, benefits, risks, and capabilities of AI tools. Acceptability of AI is defined as the extent to which counsellors, or the counselling community find the tools associated with AI relevant, suitable, and satisfactory in the counselling profession, while applicability refers to the extent of use of AI tools such as chatbots or other automated platforms for counselling services.

A structured question was developed by the researchers with four sections. Section A of the instrument was designed to collect information about the respondent's demographic information, such as gender, age, marital status, professional experience, and professional rank. Section B contained six items that were used to measure the three key variables used for the linkage. Awareness of AI among counsellors was measured with six items, and one sample item is “I *have good knowledge of how AI can be used in counselling sessions*”. Section C was for the acceptability of AI in counselling. The variable was measured with six items, and one sample item is I am open to the idea of using AI in counselling. More so, Section D, which is for the application of AI tools in counseling, w was measured with 10 items, which are *telehealth platforms, chatbots, BetterHelp, Replika, Youper, Woebot, Wysa, Talkspace, Amica, Google Cloud AutoML Tables, and Ayasdl*. The three sections B-C were measured using a four-point Likert scale of strongly agree (A), agree (A), disagree (D), and strongly disagree (SD), while section D, which is on the applicability of AI in counseling, w was measured using a five-point Likert scale that ranged from high extent (HE) to not at all (NAA).

### Validation process

The items in the instrument were validated using three experts in educational technology and three psychometric experts, who were all professors with over 10 years of experience. Their job was to quantitatively determine the suitability, precision, and representativeness of the items. The ratings of the experts aided in the computation of item content validity indices (I-CVI) and scale content validity indices (S-CVI). I-CVI ranged from 0.88–0.90, 0.81–0.89 (precision), and 0.80–0.87 (representativeness). Items that had an index less than 0.70 were trimmed off as suggested by experts (64, 65). Similarly, for the scale content validity indices (S-CVI), the range of items was 0.90–097 for suitability, 0.92–0.99 (precision), and 0.90–0.95 (representativeness). These quantitative measures helped to reduce the items from 22 to 20.

Exploratory factor analysis was carried out to determine the structure of the items with a total of 500 counsellors who were not part of the study. The instrument was administered personally by the researchers, and the respondents were allowed to respond to the instrument. After one month, the researchers retrieved all the instruments as administered, except for three that were not filled completely. Exploratory factor analysis (EFA) was performed with the principal component as the extraction method and varimax as the rotation option. A total of three factors were obtained after PER 3 for acceptability was deleted for having a factor loading less than 0.50. The three factors, as shown in Table 1, have a total explained cumulative of 70.36%. For each factor's contribution, application of AI in counselling practices contributed 15.80%, acceptability contributed 22.18%, and awareness contributed 32.39% to the total variance. The KMO test of sampling adequacy yielded a coefficient of.752, while the Bartlett's test of sphericity yielded a significant result, χ^2^(154) = 1,431.64, *p* < .001, indicating that the correlation matrix was not an identity matrix and that the sample size of 500 was adequate or sufficient for the performance of factor analysis.

**Table 1:** Exploratory factor analysis and internal structure of the scale.

Items	M	SD	ε	λ	λ^2^	Construct attributes
APP5	2.480	.546	.007	.850	.723	
APP1	2.433	.548	.007	.846	.712	
APP6	2.260	.705	.009	.837	.701	AVE = .640 Discrim = .410 *α* = .760
APP9	2.299	.457	.006	.797	635	
APP4	2.386	.486	.006	.795	.635	
APP3	2.462	.549	.007	.784	.615	
APP10	2.419	.548	.007	.776	.602	
APP8	2.212	.719	.009	.772	.595	
APP2	2.297	.457	.006	.772	.595	
APP7	2.364	.481	.006	.767	.588	
SUM	23.615	4.438	.060	7.996	6.401	
ACC6	2.384	.582	.007	.908	.824	AVE = .750 Discrim = .562 α = .760
ACC2	2.272	.510	.006	.896	.802	
ACC5	2.384	.589	.008	.854	.729	
ACC1	2.260	.516	.007	.852	.725	
ACC4	2.323	.546	.007	.821	.674	
SUM	11.626	2.420	.033	4.333	3.754	
AWR6	2.534	.693	.009	.818	.669	AVE = .603 Discrim = .363 *α* = .760
AWR5	2.498	.718	.009	.811	.657	
AWR4	2.505	.692	.009	.794	.630	
AWR3	2.532	.766	.010	.793	.629	
AWR2	2.690	.719	.009	.771	.594	
AWR1	2.644	.745	.010	.663	.439	
SUM	15.407	3.421	.046	4.650	3.618	

AVE, average variance extracted; discrim, discriminant validity; α, Cronbach alpha; λ, factor loadings.

To establish discriminant and convergent validity, the study followed the suggestion of the Fornell-Larcker criterion (66), which relies mostly on the average variance extracted (AVE) and the composite reliability measures to determine these qualities. According to the scholars, where the AVE for each subscale is greater than 0.50, such measures are accepted as adequate for convergent validity, and where the square root of the AVE is greater than the inter-construct correlation coefficient of each of the subscales, it is established that discriminant validity exists. When these occur, it is always an indication that items could separate themselves from unrelated variables (67, 68). The result in Table 1 presents the factor loadings of each item, the average variance extracted (AVE), reliability, and discriminant validity of each factor.

### Ethical consideration

In the behavioural research like survey that possess no harm or significant threat to the participant or respondents, ethical clearance can be waived according to the Nigeria Code for Health Research Ethics (NCHRC) (see https://bit.ly/3pK9ORh). However, in line with best practices, the researchers ensured that approval was obtained for this research since human participants are involved and their rights and privileges must be respected Thus, the University Ethics Committee under the Department of Quality Assurance was written to and approval was obtained (see ref: IRC/CAL/004/0766).

### Procedure for data collection

The researchers collected the data through electronic means with the help of different professionals in different universities. A total of 76 research assistants were used for this study to support the team and they were financially induced for that purpose. First, the researchers were able to identify different professionals who already are colleagues from different universities, and they were rightly informed of the exercise before the arrival of the team. The duties of this research assistants were clearly explained, chief among them was to ensure that the instrument was posted to platforms where professional counsellors are members. This was done through a zoom meeting to facilitate questions and answers from the team members. They were instructed not to send it to students' platforms nor any other platform apart where professionals are like the Counselling Association of Nigeria (CAN) platforms. A.csv file was created for responses to be obtained electronically from those who complete their responses and submit them. Compulsory options in Section A were asterisks to obtain the demographic information of the respondents in terms of their professional experiences and ranks as well as the provision of consent for the study. The administration and collation of data took ten months (March 2023 to December 2023). A total of 5,399 counsellors' responses were finally obtained for the study. A variance approach to structural equation modelling was employed in testing the hypothesized model earlier proposed. The results of the analysis are presented in the following section.

## Results

Hypothesis 1a, 2a and 3a were tested using one sample test and the result as presented in Table 2 revealed that counsellor's awareness of AI in practices (M = 15.407, S.D. = 3.402) at a 95% CI [15.315, 15.499], t(5,338) = 328.979, *p* < .001. This implies that the level of awareness among counsellors to AI tools in counseling is significantly high. The alternate hypothesis is supported for H1a. For H2a which is the level of acceptability of AI tools among counsellors, (M = 11.626, S.D. = 2.428) at a 95% CI [11.561, 11.691], t(5,338) = 349.804, *p* < .001, which indicates that counsellors' acceptability level of AI tool is significantly high. Thus, the null hypothesis for H2a is rejected. For H3a which is on level of applicability of AI tools in counselling practices, (M = 23.610, S.D. = 4.431) at a 95% CI [23.496, 23.706], t(5,338) = 389.373 *p* < .001. This is an indication that counsellors apply AI tools in their professional practices. Thus, the null hypothesis for H3a is rejected and the alternate hypothesis supported. The differentials based on professional rank and experience will be presented in multigroup analysis results.

**Table 2:** One sample *t*-test analysis of the level of awareness, acceptability and application of AI tools in counselling practices.

Variables	*N*	M	SD	SE	df	*t*-val	*p*-val	95% CI	
Awareness	5,339	15.4076	3.42213	.04683	5,338	328.979	.000	15.3158	15.4994
Acceptability	5,339	11.6261	2.42851	.03324	5,338	349.804	.000	11.5610	11.6913
Application	5,339	23.6149	4.43149	.06065	5,338	389.373	.000	23.4960	23.7338

CI, confidence interval; M, mean; SD, standard deviation; SE, standard error.

### Test of prediction

Partial Least Squares (PLS) structural equation modelling was used to examine the linkages between awareness, acceptability, and application of AI in counseling practices. More so, the effect of awareness on application through acceptability was tested using mediation analysis. Acceptability collectively explains 3.9% of the variation in counsellors' application of of AI counselling practices R^2^ = 0.039, *p* < .05. Similarly, counsellors' awareness and acceptability combined contributes 11.7% to the variation in application of AI in counselling, R^2^ = 0.117, *p* < .05

**Figure 2:**
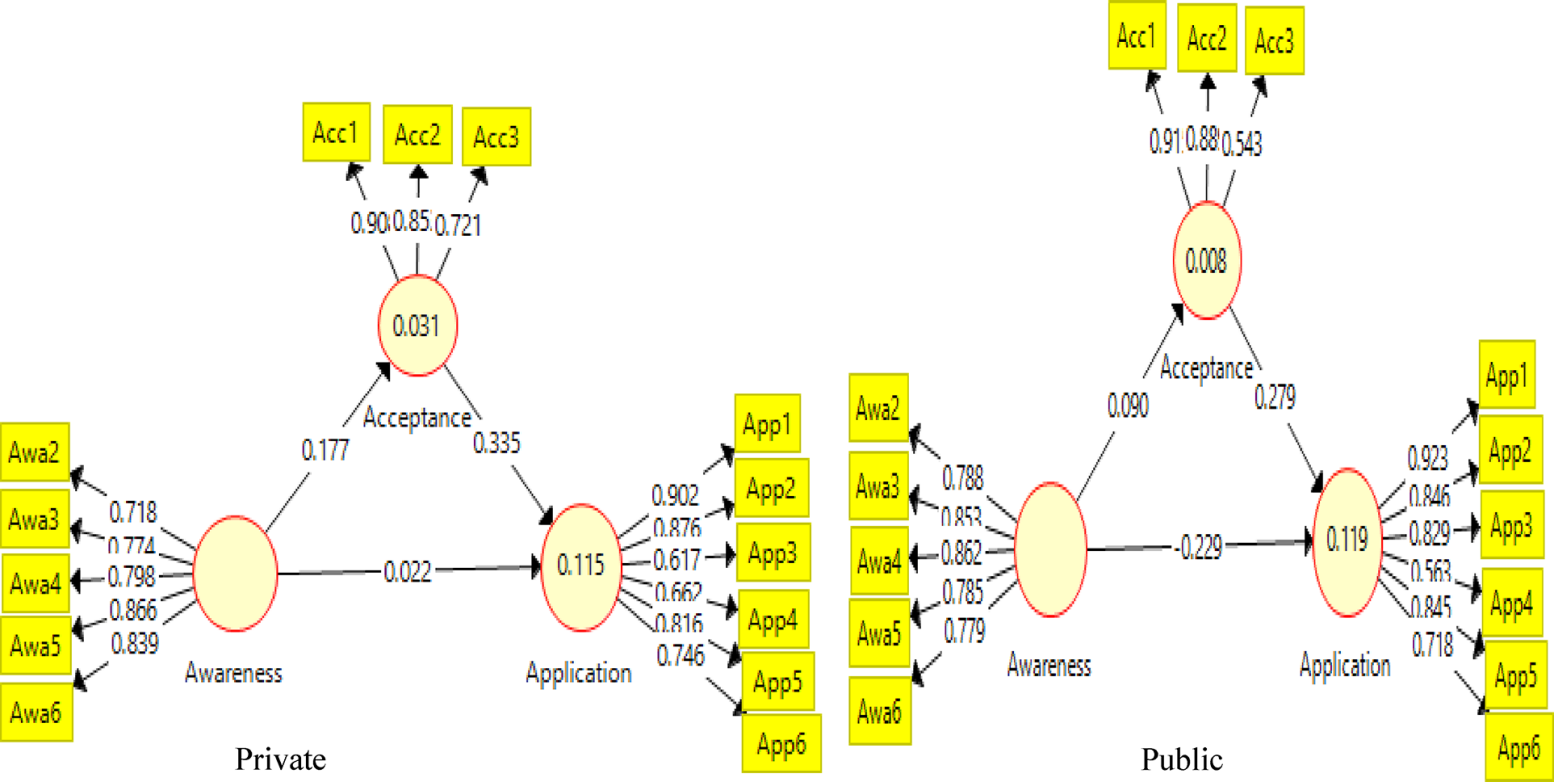
Structural equation model connecting awareness, acceptability, and application of AI in counselling practices.

The result for H1b, H1c, H1d, H2b as presented in Figure 2 and Table 3 indicates a significant negative direct effect of awareness [*β* = −.179, 95% CI (−.024, .021), *t* = 2.712, *p* < .001] on application of AI counselling practices. Therefore, H1b is rejected was supported. The result for H1c as presented in Table 3 showed a significant positive direct effect of awareness [*β* = .198, 95% CI (.167, .232), *t* = 11.66, *p* < .001] on the acceptability of AI in counselling practices. Thus, H1c was rejected by evidence. Similarly, on the direct effect of acceptance on the applicability of AI in counselling practices [H2b: *β* = 0.33, 95% CI (.295, .3661), *t* = 17.866, *p* < .001] which implies that is a positive direct effect of acceptability of AI and its application in counselling practices. Thus, the alternate hypothesis is supported. Finally, the result for H1d which is on the mediating effect of awareness on applicability of AI through acceptance of AI tools showed that [*β* = .065, 95% CI (.053, .082), *t* = 8.71, *p* < .001] which is an indication that there is a partial mediating effect of awareness on Applicability of Ai through acceptance. Thus, the null hypothesis is rejected.

**Table 3:** Direct and indirect effect of variables.

Linkages	β	95% CI	M		SD	*t*	*p*-val	Remarks
Acceptance -> Application	0.330	0.295	0.366	0.332	0.018	17.866	<.001	Rejected
Awareness -> Acceptance	0.198	0.167	0.232	0.20	0.017	11.659	<.001	Rejected
Awareness -> Application	−0.179	−0.24	0.021	−0.163	0.066	2.712	<.001	Rejected
Awareness -> Acceptance > Application	0.065	0.053	0.082	0.066	0.007	8.71	<.001	Rejected

M, mean; SD, standard deviation; CI, confidence interval.

### Gender differences in the nexus between the explanatory and criterion variables

**Figure 3:**
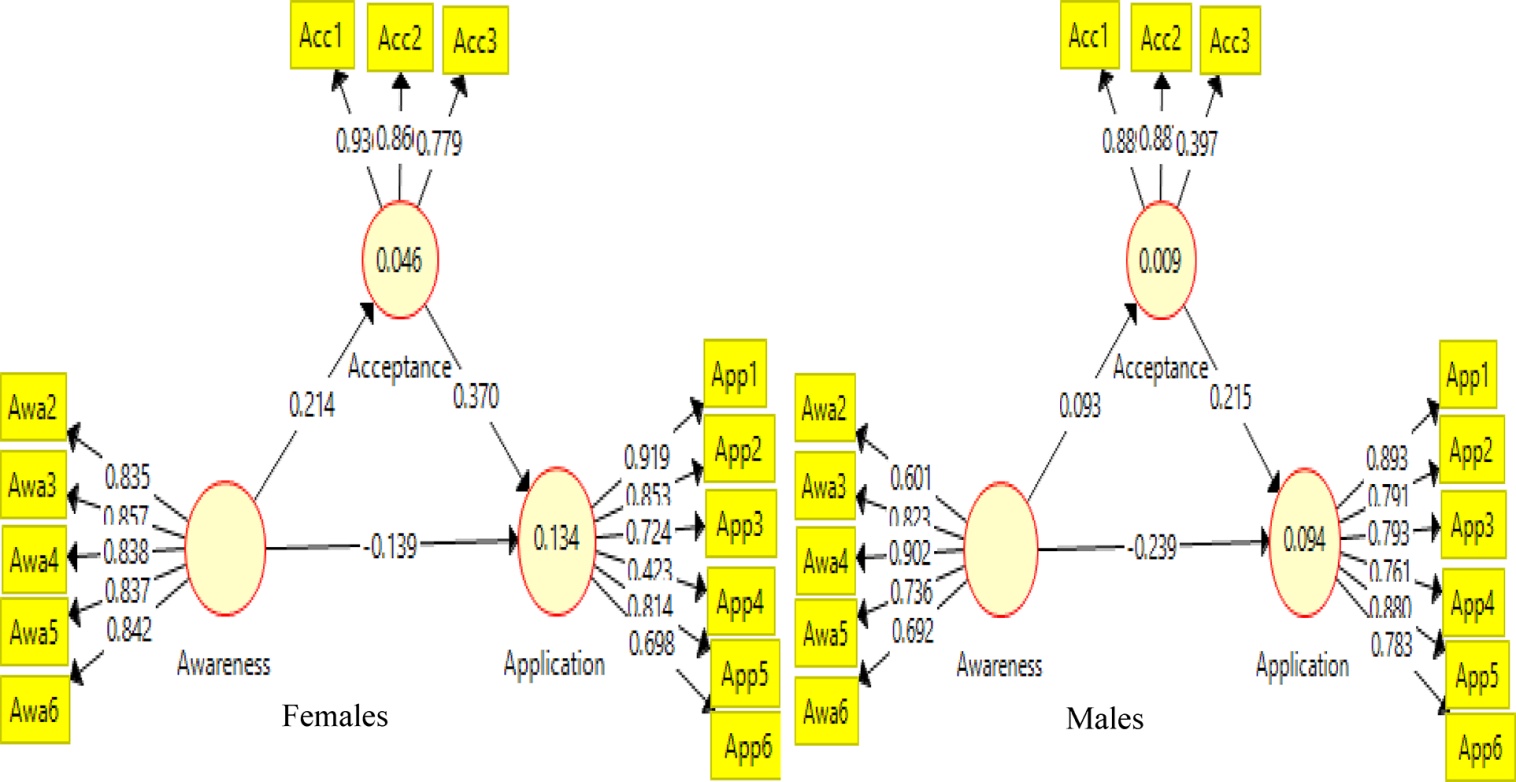
**(a)** Males. **(b)** Females.

The result as presented in Table 4 is for H4a, H4b, H4c and H4d and Figures 3A,B. The result showed that counsellors awareness significantly predicts acceptability of AI tools for counselling positively for both females (*β* = .16, *t* = 7.56, *p* < .001) and males (*β* = .27, *t* = 11.71, *p* < .001), with the effect being stronger on males. The permutation test found a significant gender difference (*δ* = −0.001, *p* < .001) in the prediction of awareness on counsellors' awareness of AI tools in counseling practices. H4a, based on the result, was rejected. Similarly, for H4b, counsellors' awareness (*β* = −.06, *t* = 0.73, *p* > .05) does not significantly predicts application of of AI in counselling for males but negatively for females (*β* = −.33, *t* = 12.24 *p* < .001), with the effect being stronger on the female counsellors than the males. The permutation test found a significant difference (*δ* = −.33, *p* < .001) in how awareness contributes to counsellors “ application of AI in counselling sessions in females than male students. Therefore, our hypothesis was rejected. Similarly, the result in Table 4 for H4c showed that acceptability significantly predicts the application of AI in counselling both for females (*β* = .29, *t* = 15.04, *p* < .001) and males (*β* = .34, *t* = 14.50, *p* < .001), with the effect being relatively stronger in males than the female students. The permutation test found a non-significant difference (*δ* = −001, *p* > .05) in how acceptability contributes to application of AI tools for counselling between males and females. Therefore, our hypothesis was sustained. H4d as presented in Table 4 further shows that acceptability mediates significantly the relationship between counsellors” awareness and application of AI research tools, both positively for females (*β* = .04, *t* = 6.39, *p* < .001) and males (*β* = .09, *t* = 9.12, *p* < .001). The mediation effect was stronger for males than for female counsellors. The permutation test reveals a significant difference (*δ* = −.149, *p* < .001) in the mediation effect of acceptance for both male and female respondents in the linkages. Therefore, H4d was rejected. The result in Figure 3 further showed that awareness and acceptability, when combined, explain 8.7% of the variance (R^2^ = .087) in female counsellors’ application of AI in counselling, while in males, both variables combined explain 17.0% of their application (R^2^ = .170). Similarly, awareness explains 2.6% of the variance in female counsellor's acceptance of AI in counselling, while for male's, it contributes 7.5% of the variance in their acceptability of AI tools. This showed that students' awareness, acceptance and applicability is stronger for male counsellors than the female counsellors.

**Table 4:** Multi group analysis based on gender.

Linkages	*B* Females	95% CI		M	SD	*t*-val	*p*	Remarks
Acceptance -> Application	0.299	0.266	0.335	0.292	0.02	15.048	<.001	rejected
Awareness -> Acceptance	0.162	0.118	0.203	0.161	0.022	7.461	<.001	rejected
Awareness -> Application	−0.061	−0.182	0.096	−0.056	0.084	0.728	0.467	accepted
Awareness -> Acceptance -> Application	0.047	0.035	0.064	0.047	0.008	6.394	<.001	rejected
Linkages	Males	95% CI		M	SD	*t*-val	*p*	Remarks
Acceptance -> Application	0.347	0.284	0.386	0.35	0.024	14.509	<.001	rejected
Awareness -> Acceptance	0.271	0.224	0.314	0.271	0.023	11.71	<.001	rejected
Awareness -> Application	−0.336	−0.382	−0.274	−0.336	0.027	12.24	<.001	rejected
Awareness -> Acceptance -> Application	0.094	0.075	0.115	0.095	0.01	9.118	<.001	rejected
Linkage	Baseline	Pairwise permutation			95% CI		*p*	Remarks
		Female	Male	Δ				
Acceptance -> Application	0.330	0.299	0.347	−0.001	−0.06	0.059	0.107	Accepted
Awareness -> Acceptance	0.198	0.162	0.271	−0.001	−0.066	0.063	0.001	Rejected
Awareness -> Application	−0.179	−0.061	−0.336	−0.005	−0.122	0.096	0.001	Rejected
Awareness -> Acceptance -> Application	0.065	0.049	0.094	−0.001	−0.027	0.025	0.211	Rejected

M, mean; SD, standard deviation; CI, confidence interval.

### Professional rank differences in the nexus between the explanatory and criterion variables

The result as presented in Figure 3 and Table 5 is for H4e, H4f, H4g and H4h. The result showed that counsellors awareness significantly predicts acceptability of AI tools for counselling positively for both females (*β* = .16, *t* = 7.56, *p* < .001) and males (*β* = .27, *t* = 11.71, *p* < .001), with the effect being stronger on males. The permutation test found a significant gender difference (*δ* = −0.001, *p* < .001) in the prediction of awareness on counsellors' awareness of AI tools in counseling practices. H4a, based on the result, was rejected. Similarly, for H4b, counsellors' awareness (*β* = −.06, *t* = 0.73, *p* > .05) does not significantly predicts application of of AI in counselling for males but negatively for females (*β* = −.33, *t* = 12.24 *p* < .001), with the effect being stronger on the female counsellors than the males. The permutation test found a significant difference (*δ* = −.33, *p* < .001) in how awareness contributes to counsellors “application of AI in counselling sessions in females than male students. Therefore, our hypothesis was rejected. Similarly, the result in Table 4 for H4c showed that acceptability significantly predicts the application of AI in counselling both females (*β* = .29, *t* = 15.04, *p* < .001) and males (*β* = .34, *t* = 14.50, *p* < .001), with the effect being relatively stronger in males than the female students. The permutation test found a non-significant difference (*δ* = −001, *p* > .05) in how acceptability contributes to application of AI tools for counselling between males and females. Therefore, our hypothesis was sustained. H4d as presented in Table 4 further shows that acceptability mediates significantly the relationship between counsellors” awareness and application of AI research tools, both positively for females (*β* = .04, *t* = 6.39, *p* < .001) and males (*β* = .09, *t* = 9.12, *p* < .001). The mediation effect was stronger for males than for female counsellors. The permutation test reveals a significant difference (*δ* = −.149, *p* < .001) in the mediation effect of acceptance for both male and female respondents in the linkages. Therefore, H4d was rejected. The result in Figure 3 further showed that awareness and acceptability, when combined, explain 8.7% of the variance (R^2^ = .087) in female counsellors' application of AI in counselling, while in males, both variables combined explain 17.0% of their application (R^2^ = .170). Similarly, awareness explains 2.6% of the variance in female counsellor's acceptance of AI in counselling, while for male's, it contributes 7.5% of the variance in their acceptability of AI tools. This showed that students' awareness, acceptance and applicability is stronger for male counsellors than the female counsellors.

**Table 5:** Multi group analysis based on professional rank.

Linkages	Asst lect-Lect II (G1)			Lect 1 -Snr Lect (G2)				Professor (G3)		
	β	*t*	*p*	β		T	*p*	β	T	*p*
Acceptance -> Application	0.605	32.72	.000	0.159		4.638	.000	−0.16	3.389	.000
Awareness -> Acceptance	−0.115	0.991	.322	−0.092		0.951	.342	−0.38	18.33	.000
Awareness -> Application	−0.359	0.997	.319	−0.331		6.686	.000	−0.31	10.92	.000
Awareness -> Acceptance -> Application	−0.070	0.991	.322	−0.015		0.852	.394	−0.06	3.146	.002
Test of hypothesis	Baseline			Pair wise permutation test						
Hypothesis	G1	G2	G3	G1 vs. G2		G1 vs. G3		G2 vs. G3		
				*δ*	*p*	*Δ*	*p*	δ	*P*	
Acceptance -> Application	0.605	0.159	−0.16	.445	.001	.764	.000	.319	.003	
Awareness -> Acceptance	−0.115	−0.09	−0.38	-.023	.703	-.496	.000	-.473	.000	
Awareness -> Application	−0.359	−0.33	−0.31	-.280	.689	-.042	.848	-.013	.715	
Awareness -> Acceptance -> Application	−0.070	−0.01	−0.06	-.055	.040	-.009	.538	-.046	.336	

Asst, assistant; Lect, lecturer; Snr, senior.

The results presented in Table 5 are for H4e, H4f, H4g, and H4h. The result for H4e revealed that the direct effect of awareness on acceptability of AI for professionals who are between Asst Lect and Lect II (*β* = −.11, *t* = .99, *p* > .05) is negatively and non-significant, and for those who are between Lect 1 and Senior Lect (*β* = .09, *t* = .95, *p* > .05), but negatively significant for those who are professors (*β* = .38, *t* = 18.33, *p* < .0015), with the effect being stronger on those who are professors. The permutation test found a significant difference, but it was stronger consistently for those between Asst. lect and Lect. II compared to the other groups in the prediction of awareness of AI tools in counselling practices to acceptability. H4e, based on the result, was rejected. Similarly, for H4f, counsellors' awareness (*β* = −.36, *t* = .99, *p* > .05) does not significantly predict Asst Lect-Lect, but it for holds significantly predict those between Lect I-Snr Lect (*β* = −.36, *t* = 6.68, *p* < .001) and those who are professors (*β* = −.31, *t* = 10.92, *p* < .001). This showed that the awareness effect on application is stronger for professors and those between Lect I-Snr Lect. This is further shown in the paired-wise permutation, even though it showed an insignificant result across the three groups. Hence, the null hypothesis is supported. For H4g, which was on the direct effect of acceptance on application, for Asst.lect-Lect II (*β* = .61, *t* = 32.72, *p* < .001), it showed a significant effect, like wise for those who are Lect I-Snr Lect (*β* = .15, *t* = 4.64, *p* < .001) and Professors (*β* = −.16, *t* = 3.389, *p* < .001). This implies that all three groups have strong acceptability for the applicability of AI in counselling practices. Thus, H4g is rejected. For the mediating effect of acceptance on the linkage between awareness and application, for those who are Asst Lect-Lect II, it is negatively insignificant (*β* = −.07, *t* = .99, *p* > .05), and negatively insignificant for those who are Lect-I-Snr (*β* = −.01, *t* = .85, *p* > .05), but negatively significant for those who are professors (*β* = −.06, *t* = 3.146, <.001). The pair-wise permutation showed that even though it is stronger for professors, it does not vary strongly across all the groups. Thus, the hypothesis is retained.

### Assessment of outer model

The baseline model is presented in Figure 2 and provides the item loadings for each variable. The items loaded were appropriate except for APP 9 (.395) and APP 4 (.422) in the application that were below the .70, which is kept as a desirable benchmark (69). The items were not deleted since other assessment criteria were met (70). In Figure 3, the outer loading for gender was examined, and the results revealed that the loading of two items ranged appropriately except for APP 9 (.244) for the males. For professional ranks, item loading was appropriate for those who are Asst Lect-Lect II, except for some items that loaded poorly (APP 9-.516; APP 4-.515, and Awr1-.167). For those who are Lect I-Snr lect, item loading has some poor items loading in the model (APP 10-.531, AWR 1-.219, AWR 2-.449, and AWR 5-.279). Importantly, some items loaded poorly into the latent construct, such as items in APP and AWR. The removal of these items, even though they were poorly loaded, will affect the reliability of the measures, but the items for awareness may not be suitable for the professional counsellors since they loaded poorly for both gender and rank.

### Convergent and divergent validity

The Fornell-Larcker criterion was used to determine the validity of the measure, which uses the average variance extracted (AVE) for the measurement models in the study. The entire variables had an AVE that was above 0.50 except awareness for professors, which was relatively below 0.50. In Table 6, since all the variables were above 0.50, it implies that AVE has been achieved. Similarly, for the two groups, gender and professional rank, the AVE score was above 0.50, indicating that there is convergent validity for the measures but not for awareness for the three groups in the professional ranks. Similarly, discriminant validity, which is the square root of the AVE, must always be greater than the coefficient of the correlation among variables. The result in Table 7 presents empirical evidence of the discriminant validity of the constructs.

**Table 6:** Convergent validity of measures.

Variables	Baseline	Gender		Professional rank		Professor
		Male	Female	Asst Lect-Lect II	Lect I-Snr	
Acceptability	.782	.707	.849	.726	.705	.788
Awareness	.531	.498	.659	.663	.643	.690
Application	.584	.621	.606	.367	.543	.342

**Table 7:** Discriminant validity of the measures.

Variables	Baseline			Males			Female			Asst lect-Lect II			Lect I-Snr Lect			Professor		
Acceptability (1)	0.61			.50			.72			.52			.49			.62		
Awareness (2)	0.11	0.28		.15	.24		.18	.43		.21	.43		.32	.41		.32	.47	
Application (3)	−0.121	0.20	0.34	−.21	.18	.39	.21	−.11	.36	.10	−.12	.13	.22	−.24	.29	.21	.25	−.11

HTMT. The Obtained values of HTMT values were all less than 0.90, implying that discriminant validity was evidentially achieved for the population.

### Reliability

The reliability was ascertained using the Cronbach alpha and composite reliability measures. The estimates are presented in Table 8, and the result showed that virtually all the measures are above 0.70 except for awareness under professional ranks, which could possibly be a human factor that factors in error in all forms of measurement. However, these minute discrepancies do not invalidate the findings of the study.

**Table 8:** Composite and cronbach alpha reliability estimates.

Variables	Baseline		Gender				Programme type				Professor	
	α	CR	Males		Females		Asst Lect-Lect II		Lect I-Snr Lect			
			α	CR	α	CR	α	CR	α	CR	α	CR
Acceptability	.930	.947	.896	.923	.905	.966	.906	.929	.910	.922	.948	.960
Awareness	.925	.914	.915	.898	.949	.950	.942	.950	.942	.946	.909	.953
Application	.864	.894	.880	.908	.877	.902	.588	.701	.588	.688	.506	.610

α, Cronbach alpha; CR, composite reliability.

## Discussion of finding

The study was carried out to examine the mediating effect of acceptance of AI in the nexus between awareness and application of AI in counselling practices. Variables like gender and professional rank were engaged to examine the variations among these groups. Based on the findings, the discussion is presented in the preceding sections.

### Awareness and application of AI in counselling practices

The research findings obtained from this study were that counsellors' awareness is a strong predictor of the application of AI in counselling practices. This holds true but is stronger for females than it does for males, as well as stronger for those who are professors and Lect 1-Snr Lecturers. In other words, how counsellors apply AI in counselling is determined by their level of awareness of the tools. Counsellors who have a higher level of awareness are more likely to utilise it in their counselling sessions, especially as the results have further shown that gender and professional rank are determining factors in this nexus. More so, those who are advanced in rank in the profession, like those within Lect I to professors, are more aware of this linkage.

The findings of the study, especially as they underscore the nexus between awareness of AI and its application among professors, could be due to the fact that professors are senior citizens in the profession who may have accumulated all forms of experiences via workshops, training, and exposure to AI-related trainings, and this may have contributed to shaping their perception and readiness to apply AI tools into their counselling profession. Professors and senior lecturers are often at the forefront of research and innovation in their respective fields. Their professional responsibilities may involve conducting research, publishing scholarly articles, and presenting at conferences, all of which contribute to their knowledge base and awareness of cutting-edge developments in counselling, including advancements in AI technology. As such, they may be more inclined to explore and incorporate AI tools into their counselling practice as a means of staying current and enhancing the quality of care provided to clients.

The findings also highlight the dynamics of demographic factors, which is an indication to initiate intervention programmes and educational initiatives that will ensure that all professionals, irrespective of their rank and gender, are helped to acquire the right awareness of these modern tools so as to foster a more inclusive and effective integration of AI technologies in counselling practices. Males' exhibition of a stronger effect in the nexus could not be unconnected to the fact that in Africa, most of the activities that males are allowed to do could be different from what women do, and this could account for why they are more aware than women in the application of these facilities. The finding is in line with that of previous researchers who have found that males are more exposed to and aware of new technologies than females, probably because of their insatiable exploration of devices in career and professional developments (71, 72).

Similarly, regardless of gender and professional rank, the result further showed that awareness is a strong predictor of the application of AI in counselling practices. This result underscores the universal importance of awareness as a precursor to the integration of AI in counselling, transcending gender differences. This is because counsellors who are well informed about new arrivals and the import of AI in their professions are better disposed to apply those technologies for efficient and effective client satisfaction, which is key in the counselling profession. This finding aligns also with previous studies that have shown that awareness of new technologies like AI and various LLM tools affects the pattern and way things are done in different sectors (64, 71, 72).

### Awareness and acceptability of AI in counselling practices

The research findings obtained from this study were that counsellors' awareness is a strong positive predictor of the acceptability of AI in counselling practices. This holds true for both males and females, but it is stronger for males than it is for females, as well as stronger for those who are professors compared to other groups of respondents. In other words, the findings suggest that the acceptability of AI in counselling sessions depends on the level of awareness. In this context, it means that awareness has a strong role to play in whatever counsellors may do with these modern tools in their profession. This further shows that even though awareness is important, this effect is stronger for professors who have had enough experience and exposure over time. However, irrespective of sex, the acceptability of these tools in the profession is a function of their awareness level.

This finding could be unexpected and may offer another deeper exploration into what shapes this nexus among professionals. However, awareness and acceptability of AI in counselling practices are germane in that no one accepts what he does not have adequate knowledge of. It is important that for one to accept a tool that may not have been used in a sensitive profession like counselling where ethical issues are deeply involved, there should be adequate knowledge so that bridges in terms of confidentiality and informed consent, among others, will not be taken for granted. The findings, according to Stina (36), also underscored the multifaceted nature of the counsellors' jobs, which stand on a tripod: counsellor, client, and complain (issue).

The findings indicating that counsellor awareness and acceptability of artificial intelligence (AI) in counselling practices are stronger for males than females prompt an exploration into potential underlying factors contributing to this gender disparity. One possible explanation for this finding could also be due to the fact that social norms and values, as well as the stereotypes in Africa regarding gender and information and communication technology, In most cultures, males are more allowed to adopt ICT, while females are limited due to cultural biases that limit women's engagement in ICT. Similarly, they may be disposed to seeking mentorship that female counsellors may be afraid of, as well as seeking professional development in AI-related issues. Thus, male counsellors may be more prone to using these tools, and this may have warranted a higher level of awareness and acceptability of AI than female counsellors. This is in line with previous studies that have found that males are more inclined to ICT than females (11, 12, 59, 60, 73). However, other studies contradict this finding as well (58, 74).

The research findings obtained from this study were that counsellors' acceptability is a strong positive predictor of the application of AI in counselling practices. This holds true for both males and females, but it is stronger for males than it is for females, as well as for all the groups in professional ranks. The result further implies that, irrespective of gender and professional rank, the application of AI tools is linked to their acceptance of these tools, which of course could depend on different factors. This unexpected finding prompts a deeper examination of the underlying factors contributing to such discrepancies in acceptability. The rationale for this outcome could be that most males seek professional mentorship that most females, because of security, may not be exposed to, as well as social norms that may place them in a disadvantaged position. This may have increased the level of acceptance and application of AI among male counsellors. Additionally, differences in exposure to and training in technology-related fields may also play a role in shaping gender disparities in acceptability. The same explanation applies to professors and senior lecturers adjudged to be stronger in the acceptance and application of AI, namely that they are more exposed to training, facilities, and support that have exposed them to AI-related facilities than others in the profession, and this facilitates their acceptance and application level of AI in their practices. This finding is not different from what other previous researchers have found, even though some studies focused more on education generally (45–47).

The result for the mediating effect of awareness on the applicability of AI through acceptance of AI tools showed that acceptance of AI is a positive mediator in the nexus because of awareness and application of AI in counselling practices. The result further showed that the mediating was stronger for males than females, as well as stronger for those who are professors, even though it was negative. The findings of the study could likely be due to the fact that awareness is a foundation for applying these tools. However, the result has proven that even though awareness can directly affect the application of AI, such an effect is stronger when there is a strong acceptance of these tools. One must take into cognizance that counselling involves more humans who may want to even relate to technology. It takes a counsellor who understands the rubric and dynamics of these tools to first apply them and then convince the clients to follow the prescription and direction as provided by these AI tools. The findings of the study further showed that, irrespective of gender and professional ranks, acceptance as a mediating factor is sine qua non. This finding could also be due to the fact that both sexes and ranks understand the importance of these tools in modern counselling programmes. The findings of the study are not different from what other previous researchers have found in their studies (61, 75).

Broadly speaking, the finding of this study means that AI acceptance, as a mediator, significantly altered the effect of awareness, reducing the likelihood that their positive awareness would lead to the application of AI in counselling. The finding can be explained because counsellors who accept these tools may initially see them as valuable tools for their job. However, if their acceptance is very stringent and goes with best practices, they may reconsider applying it to aid their jobs (76).

### Limitation of the study and suggestion for further studies

The study, like other studies, especially the survey, is not free from inherent limitations. First, the study does not look at academics generally but at specific professionals, like the counsellors with the Nigerian tertiary institutions, thereby excluding other professionals that may not be within the purview of tertiary institutions. Thus, it may not be difficult to absolutely apply this finding to alternative platforms like psychiatric settings or psychological professions. To rectify this deficiency, future investigations should broaden their scope to encompass professionals outside the tertiary institution as well as professional psychologists, thereby facilitating a comprehensive assessment of AI with respect to mental health and counselling practices. More so, since it is cross-sectional bias that depends on self-reports, the susceptibility of this study to respondents' biases and prejudices is note-taking. This approach poses the risk of respondents inaccurately reporting their experiences. The incorporation of alternative methodologies, such as observational techniques, could enhance the study's reliability and objectivity. It is imperative to highlight that these identified limitations, notwithstanding, do not render the study's findings invalid or inconsequential. On the contrary, the present study has provided valuable information to the existing body of knowledge on awareness, acceptance, and application of AI in counselling among professional counselors. Thus, further research is plausible to add, update, refine, or expand upon the scope, weaknesses, and strengths of this study.

## Conclusion

This study examined the nexus between awareness and application of AI through acceptance of these tools in counselling practices. The findings underscore the significant role of perception in shaping counsellors' application of AI tools. Interestingly, the study showed that counsellors' awareness is a strong positive predictor of the acceptability of AI in counselling practices. This holds true for both males and females, but it is stronger for males than it is for females, as well as stronger for those who are professors compared to other groups of respondents. Similarly, a counsellor's acceptability is a strong positive predictor of the application of AI in counselling practices. This holds true for both males and females, but it is stronger for males than it is for females, as well as stronger for all the groups in the professional ranks. A counsellor's awareness is a strong positive predictor of the acceptability of AI in counselling practices. This holds true for both males and females, but it is stronger for males than it is for females, as well as stronger for those who are professors compared to other groups of respondents. The result for the mediating effect of awareness on the applicability of AI through acceptance of AI tools showed that acceptance of AI is a positive mediator in the nexus because of awareness and application of AI in counselling practices. Overall, this study contributes valuable information to the existing literature regarding the interplay of awareness, acceptance, and application of AI in handling clients' issues during therapeutic sessions. It emphasises the importance of fostering higher awareness and acceptance of AI among counsellors, especially younger professionals, as a potential counterbalance to traditional practices. The study also underscores the need for educational institutions, professional bodies, and policymakers to address these issues and promote ethical use of AI in the rapidly evolving landscape of artificial intelligence in counselling practices. Hence, as Nigeria strives to fully integrate AI into the educational curriculum, it is imperative not to overlook the necessity of educating counsellor trainees and those in the profession on the potential benefits of AI for their professional jobs. However, measures to check abuse must also be put in place.

## Ethical consideration

The study, however, was a survey that did not mean any harm to the participants because they were not subjected to any treatment. According to the Federal Ministry of Health (77), “ethical clearance can be waived”. Section A of the questionnaire provides an explanation of voluntary participation in the survey. Those who indicated willingness to participate in the study ticked the box and provided written consent for participation in the study, while those who were not interested did not tick the instrument because it could not be submitted. The respondents were also told that the information provided would be treated with confidentiality and that the data would be anonymized so that no third person could access it. They were also informed that the results obtained from the responses would be published in a new journal.

## Data Availability

The raw data supporting the conclusions of this article will be made available by the authors, without undue reservation.
